# Chemismus of the Stomach*Chemismus: The aggregate of chemical action appertaining to the functions of the stomach. 2nd. Lactic acid is the result of fermentative action.

**Published:** 1897-04

**Authors:** Frank B. King

**Affiliations:** Houston, Texas


For the Texas Medical Journal.

### *CHEMISMUS OF THE STOMACH.

BY FRANK B. KING, M. D., HOUSTON, TEXAS.

[Read before Houston District Medical Society.]

AS TO HCL: As to the digestive ferment. The stomach is the fire-box to our organism; the still house to many, the chemical laboratory to all; the secretory absorbing sterilizer and expulsive organ. (a) A fire-box, because, during the act of digestion, the heat is generated by virtue of the chemical action taking place. (b) Distillation is the act of eliminating the more volatile elements on the one hand, and the fermentation of food on the other; the former, the physiological process, the latter, pathological. (c) The chemical laboratory, by reason that chemical action never ceases so long as food remains in the stomach. A separator and appropriator of all assimilable substances. Example: We give a lethal dose of any toxic drug. Does the stomach assimilate it? No; but at once there is an effort to expel it by emesis. Otherwise we have chemical incompatibility at once formed by the HCL, gastric ferment, or mucus, or all combined, which stop the absorption.

---

*CHEMISMUS: The aggregate of chemical action appertaining to the functions of the stomach. 2nd. Lactic acid is the result of fermentative action.

For our purpose, we will consider the stomach first, as to the mucous coat; 2nd, as to the muscular.

1st. A: The mucous coat has for its functions the secretion of the digestive fluids and ferments. HCL is transuded from the entire mucous coat. Above the level of the great cul-de-sac we have lactic acid formed. On a level and below the great cul-de-sac are seated the tubular glands which contain in their interior, the pepsin corpuscles. In the lower third of the pylorus portion, the glands are deprived of the corpuscles, and have for its functions the secretion of mucus.

2nd. A: The muscular tunic has for its functions the agitation of the alimentary mass, to impregnate it thoroughly with gastric juice—this is mechanical. The rythmical movements are produced regularly and continuously from left to right; that is to say, from the greater curvature to the pyloric extremity. The pyloric orifice opens six to eight times per minute. Nothing passes this port-hole without being reduced to a semi-liquid by the stomach. It is said that the aliment remains in this cavity from two to three hous; this, however, is a mooted question. In my observations, after over one thousand stomach washings, I have rarely found the stomach empty after five or six hours. When, at a proper moment, the contractions are augmented, and forces the contents of the stomach into the intestines. Physical action of the gastric juice is a chemical act from beginning to end. The mucus begins to secrete HCL so soon as the first act of digestion is commenced, namely, mastication.

As the alimentary bolus passes into the cardiac end of the stomach, it comes in contact with an acid solution, being an alkali mass having been thoroughly impregnated with the salivary secretions, neutralization of the alkali by HCL is begun. All albuminous substances, fibrin, casein, etc., are coagulated by HCL. The carbo-hydrates having begun disintegration by the action of ptyalin, which has transformed them into dextrin and glucose. A further change here is brought about. Lactic fermentation is set up, and we have lactic acid. The further action of HCL on the nitrogenous elements, we have sarco-lactic acid. In the first period of digestion we always find lactic acid, say during thirty minutes, by Uffelmann's test. The further increased quantity of HCL and increased elimination of heat, by reason of the chemical changes—"heat is always the result of chemical action"—the sterilizing effect of HCL is obtained which stops the lactic acid fermentation; the gradual and complete disappearance of lactic acid half an hour after meals, we have no further action on the carbo-hydrates in this organ. The HCL coagulates fibrin, casein, in fact the albuminous substances generally, then softens, and finally liquifies them. This acid solution of the albuminous products of digestion is called syntonin; this substance is soluable in acid, and dilute acid solutions are precipitated by alkalies.

At this stage of digestion we have poured from the tubular glands in the second division of the stomach the active ferment, pepsin, which is active only in acid solutions, transforming this acid solution of syntonin into pepton. This marks the last chemical act of stomach digestion. This acid solution of pepton is soluble in water, in neutral solutions, in acid solutions, and in alkali solutions. The non-precipitation of the albuminoids by neutralizing the gastric contents is evidence of complete digestion in the stomach. A solution of the peptones above mentioned is rapidly diffusible through animal membrane; it represents the assimilatable form of albuminous substances, and is only precipitated by alcohol in excess.

Between albumen at the beginning, and peptones at the end of the process of albuminous digestion, there exists certain intermediate bodies which are called collectively,—albuminosis. Of these we have noticed syntonin and peptone; pro-pepton we will only mention, is the result of the disentegration of vegetable albumen.

In conclusion, I wish to state most emphatically that pepsin is always present in the act of digestion whenever HCL is present, in any quantity, and its use in stomach trouble is based upon a misconception of facts.

